# The levels of essential nutrients and nonessential metals in different varieties of teff produced in Hidabu Abote Woreda, Oromia, Ethiopia

**DOI:** 10.1002/fsn3.4075

**Published:** 2024-03-04

**Authors:** Moges Kebede Seyoum, Girma Regassa Fayissa, Girma Selale Geleta

**Affiliations:** ^1^ Department of Chemistry, College of Natural Sciences Salale University Fiche Ethiopia

**Keywords:** essential nutrients, ICP‐OES, nonessential metals, teff grain, wet digestion

## Abstract

Ethiopia is the world's largest producer of teff (*Eragrostis tef* Zucc). This research was intended to determine the levels of mineral nutrients (metals and nonmetals) and nonessential metals in the grains of teff. Following the optimization of the method, the samples were wet‐digested using reagents (5‐mL HNO_3_ and 1‐mL HClO_4_) at a temperature of 230°C for two and a half hours and analyzed using inductively coupled plasma–optical emission spectrometry. Under the optimum procedure, the coefficients of determination (*R* ^2^) ranged between .9980 and .9999. The limits of detection and limits of quantification were in the range of 0.036–14.49 and 0.111–43.93 mg/kg, respectively. The recovery ranged from 80.72% to 107.79%, indicating that the method was accurate. The optimized and validated method was applied to quantify the levels of analytes in the teff samples. The overall mean concentrations of the analytes in the three varieties of teff samples were determined (mg/kg) to be in the order P (3890–4853) > K (3040–3784) > Ca (1906–1959) > Mg (1402–1698) > Fe (128–305) > Mn (64–127.8) > Na (50–136.5) > Zn (19.8–27.3) > B (1.8–21.9) > Cu (4.17–6.9) > Ni (2.6–4.05) > Hg (1.8–4.0) > Pb (0.048–3.7) > Cd (0.012–2.09) > As (0.02–0.24), with a % RSD ranging between 0.017 and 11.1. The results revealed that teff grains are a good source of minerals and contain a significant amount of toxic elements, such as Cd, Hg, Pb, and As.

## Graphical abstract

Optimizing the regent volume and ratio, temperature, and digestion duration led to the development of wet digestion sample preparation. Based on this, the optimal condition for sample preparation was found to be regents (a combination of 5‐mL HNO_3_ and 1‐mL HClO_4_) at 230°C for 150 min. Inductively coupled plasma optical emission spectroscopy (ICP‐OES) was used to analyze teff samples for 17 analytes (both essential and nonessential) once the established method had been validated.

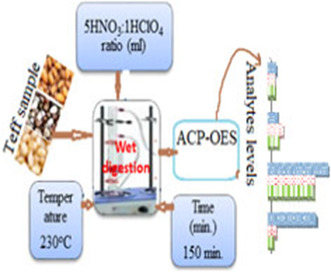

## 1 INTRODUCTION

Ethiopia has long been the home of the cultivation and consumption of teff (*Eragrostis tef*). Ethiopian teff is a popular grain for food and a native of Ethiopia. Teff is an essential crop for maintaining the food supply in Ethiopia and the East African Highlands (Girma & Meareg, 2017). The major staple food “injera,” made from teff, constitutes more than 66% of the diet in Ethiopia. Injera is a type of flatbread that is fermented, naturally leavened, and native to Ethiopia. However, injera quality can vary depending on the processing steps used, even if the same variety of teff is used (Bikila et al., 2024). Foods from multiple grains at some defined mixing ratios are more likely to contain increased or multiple or essential nutrients than foods from a single (mono) grain. Teff grain is becoming more popular in baby meal mixes with other grains, such as soybean and chickpea, because of its high mineral content (Heiru et al., 2019; Tura et al., 2023). Teff is also a promising ingredient for bread production (Harth et al., 2018). Teff, named for its small size, which makes it easy to lose and challenging to retrieve when spilled, signifies a “lost seed” in Amharic (Abraham, 2015). There are several varieties of teff in Ethiopia. Among these, the three major categories can be identified as white, red, and mixed (Baye et al., 2014).

In addition to being a great source of iron, teff contains high concentrations of calcium, potassium, and other vital elements that are present in equivalent quantities in other grains. Previous research (Abebe et al., 2007) revealed that teff flours contained greater concentrations of several important metals (Ca, Fe, and Zn) than did other common cereals. Research has indicated that the root systems of cereal crops can take up nutrients and heavy metals from soil. The extent of uptake varies among different cereal species, with some crops exhibiting greater mineral and heavy metal accumulation than others. Teff is a crop that can absorb minerals from the soil. Essential and nonessential compounds adsorb on the surface of roots, travel through roots passively, and diffuse through translocating water streams (Shahid et al., 2017).

Mineral nutrients enhance plant productivity by supporting physiological processes such as transpiration, respiration, and photosynthesis. In addition to the various functions that minerals perform in both humans and plants, the mineral contents of plants contribute to their increased nutritional value. The bioavailability of some minerals may be affected by the presence or absence of other minerals. In other words, minerals work in concert with one another to be available and support bodily functions. Likewise, the transfer of hazardous metals to the edible portions of plants is of particular concern because it can lead to health problems through the food chain. Long‐term exposure to high concentrations of these hazardous metals can cause damage to internal organs and increase the risk of developing cancer, among other health issues. Toxic metals and essential minerals need to bind with transporters at absorption sites (roots) to be absorbed. Due to their similar chemical characteristics, many hazardous metals compete with essential metals for the same binders or transferors during the absorption and translocation processes.

A few papers have investigated metals found in teff samples (Abraha, 2021; Abraha et al., 2020; Habte et al., 2020; Zeleke, 2009). The current work is unique in that it covers a large number of analytes, including metallic minerals, nonmetallic minerals, and toxic metals. At the national level, Hidabu Abote was placed fifth among the top 25 districts that produce teff; it was ranked first in the North Shoa administrative zone and fourth in the Oromia region (Negussie, 2020). Despite being a recognized teff‐producing region, no study has been performed on the amounts of mineral nutrients and nonessential metals in the teff harvested in that region. Thus, the goal of the current study was to use inductively coupled plasma–optical emission spectroscopy (ICP‐OES) to assess the concentrations of mineral nutrients and nonessential elements in the three types of teff from Hidabu Abote Woreda.

## 2 MATERIALS AND METHODS

### 2.1 Research design

The three varieties of teff were collected from mill houses. After collection, the teff samples were transported to the laboratory for sample preparation and analysis. Once the wet digestion procedure of the sample preparation process was developed, instrumental analysis was carried out utilizing the inductively coupled plasma–optical emission spectroscopy (ICP‐OES) technique. Various parameters, including temperature, digestion time, and the regent (acid) ratio, were optimized for instrumental analysis. After validation of the optimized method, the contents of the analytes were determined in the three samples of teff. The obtained data were discussed and interpreted in comparison to the previous results.

### 2.2 Description of the study area

**FIGURE 1:**
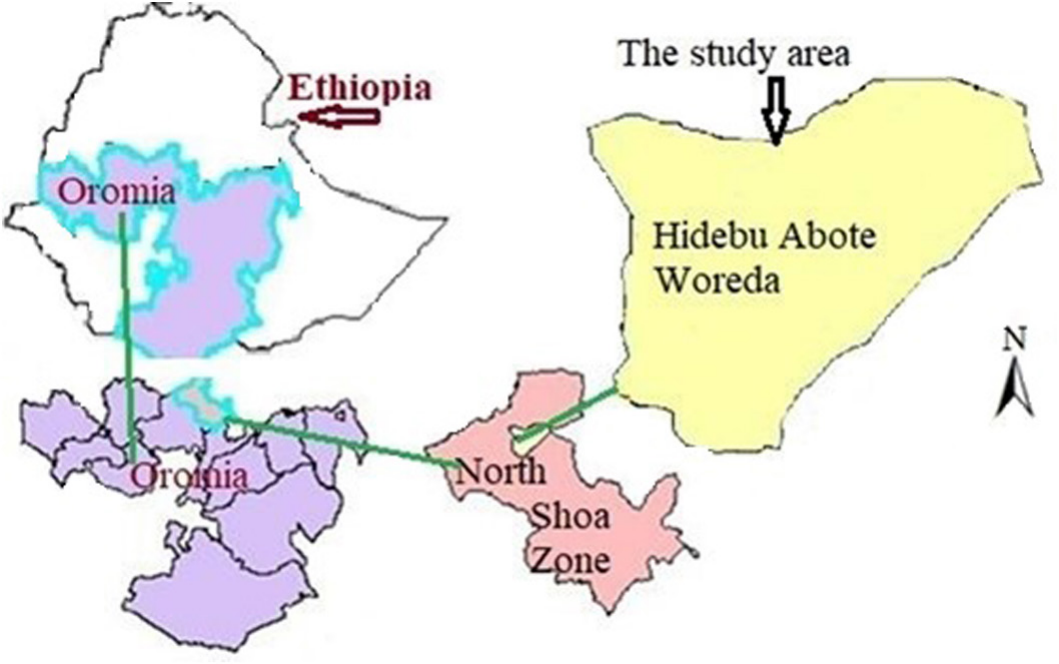
Geographic location of the experimental sites (Dereje et al., 2020).

The Hidabu Abote is located between latitudes 9°48′30″ and 10°4′40″ N and longitudes 38°24′00″ and 38°40′12″ E. It is approximately 147 km north of Addis Ababa, the capital of Ethiopia, and 42 km from Fitche, the seat of the zonal region. In the woreda, the main crop varieties grown are beans, teff, sorghum, wheat, barley, maize, and peas. The Hidabu Abote is located between 1160 and 3000 m above sea level. The woreda boasts a variable climate as well, with an average yearly temperature ranging from 13 to 20°C and an average annual precipitation ranging from 800 to 1200 mm. There are three types of soil: 14% sandy, 51% clay, and 35% silty. The district covers 50,870.39 ha in total. A total of 32,917 (64.7%) of the total area is agricultural land (Negussie, 2020). Figure 1 shows the location of the study area (Hidabu Abote).

### 2.3 Chemicals and reagents

All the chemicals and reagents used were of analytical grade. Teff samples were digested using 70% HClO_4_ (Research‐Lab Fine Chemistry Industries Mumbai 400002 [India]) and HNO_3_ (69%–70%) (Supreme Enterprises Cantt, Germany). Intermediate standard solutions were made using stock standard solutions (Buck Scientific Puro‐Graphic, USA) of each mineral nutrient and nonessential metal. Throughout the experiment, deionized water was utilized for sample preparation, dilution, and rinsing.

### 2.4 Instrument and apparatus

A temperature‐controlled digital oven (Digit Heat, J.P. Selecta, Spain) was used to dry the cleaned teff samples. Using a mortar and pestle, the teff samples were mashed and homogenized. A computerized analytical balance (Mettler Toledo, Model AG 204, Switzerland) was used to weigh the teff samples. A 250‐mL round‐bottom flask fitted with a reflux condenser was used in the Kjeldahl apparatus (Gallenkamp, England). Measuring cylinders, pipettes, beakers, volumetric flasks, and digestion tubes were used. The samples that had been digested were stored in a refrigerator (Hitachi, Tokyo, Japan) until examination. The metal concentrations were measured with inductively coupled plasma–optical emission spectrometry (SPECTRO ARCOS FHS12, Kleve, Germany).

### 2.5 Sample collection

White, mixed, and red teff samples were collected separately in polyethylene plastic bags from millhouses located in Hidabu Abote Woreda. There were nine millhouses in Hidabu Abote Woreda. These millhouses also serve as stores for supplying teff grain to town residents and other users from surrounding areas. The teff samples that were harvested from Hidabu Abote Woreda were collected repeatedly from March to July by asking whether they were from Hidabu Abote. The collected teff grains of the same type were pooled together and mixed well to form a composite sample.

### 2.6 Sample preparation

Teff samples collected from the mill houses were kept in polyethylene bags. The teff grains were separated from impurities by sifting, winnowing, and screening to eliminate the husk and other nonteff pollutants. After being cleaned with tap water as needed to remove any remaining dust, the sample was rinsed three times with deionized water. To obtain a constant mass, the samples were dried in an oven at 105°C. The dried samples were ground well using a mortar and pestle and kept in a desiccator until digestion. A 0.5 g of aliquot from each of the three varieties of teff samples (three from each bulk sample) was used for final digestion.

### 2.7 Digestion of teff samples

Wet acid digestion is a method that is often employed to remove free metal ions from complex plant matrices. Adjusting the digestion parameters, including temperature, duration, reagent type, and reagent‐to‐volume ratio, is the basis of this approach. Based on this, a Kjeldahl digestion device was used to digest 0.5 g of well‐powdered teff sample for 150 min at 230°C using a mixture of 5.0 mL of HNO_3_ (69%–72%) and 1 mL of HClO_4_ (70%). The digested solution was allowed to cool for 20 min at room temperature. Next, deionized water was added, and the mixture was shaken to dissolve any precipitate. After that, 125‐mm‐diameter filter paper was used to filter the mixture into a 25‐mL volumetric flask. For every sample, three digestions were performed. The reagent mixture was digested using the same process as before, and the resulting blank solutions were subsequently diluted to a volume of 25 mL using deionized water. The digested samples were stored in a refrigerator until further analysis via ICP‐OES.

## 3 RESULTS AND DISCUSSION

### 3.1 Optimization of wet digestion

The method for the digestion of teff samples was evaluated using variable parameters such as the volume and ratio of mixtures of HNO_3_ and HClO_4_, digestion time, and digestion temperature. Concentrated perchloric acid is a powerful oxidizing agent when heated. However, nitric acid was mixed with perchloric acid to dilute the former due to the potential for explosion. Additionally, this ensures that the molecules that are easily oxidized are broken down by a low‐temperature interaction with nitric acid before perchloric acid begins to exert its oxidizing activity at 160°C (Regassa & Chandravanshi, 2016). The teff sample was digested until a clear, colorless sample solution was obtained, which was appropriate for ICP‐OES analysis. As shown in Table 1, an optimum condition with a shorter digestion time, a lower reagent volume, a smaller ratio of HClO_4_ acid, and a lower temperature was chosen. Thus, the optimal digestion procedure for 150‐min duration for complete digestion of 0.5 g of teff powder, 5 mL of HNO_3_, and 1 mL of HClO_4_ (Table 1) at 230°C was used throughout the analysis.

**TABLE 1:** Optimization of digestion procedures for 0.5 g of teff sample.

Trial	Used regents	Volume ratio (mL)	Temperature (°C)	Digestion time (min)	Observation
1	HClO_4_:HNO_3_	1:1	180	120	Deep yellowish and turbid
2	HClO_4_:HNO_3_	1:2	180	120	Deep yellow with suspension
3	HCLO_4_:HNO_3_	1:3	180	120	Deep yellowish with cloudy suspension
4	HClO_4_:HNO_3_	1:4	180	120	Light yellowish with few suspension
5	HClO_4_:HNO_3_	1:5	180	120	Light yellowish and clear
7	HClO_4_:HNO_3_	1:5	180	150	Light yellow and clear
9	HClO_4_:HNO_3_	1:5	200	150	Almost colorless and clear
10	HClO_4_:HNO_3_	1:5	230	150	Clear and colorless (optimum)
11	HClO_4_:HNO_3_	1:5	260	120	Clear and almost colorless
12	HClO_4_:HNO_3_	1:4	260	150	Clear and few suspension
13	HClO_4_:HNO_3_	2:4	260	150	Clear and colorless
14	HClO_4_:HNO_3_	2:4	230	150	Clear and very few suspension
15	HClO_4_:HNO_3_	1:5	200	180	Clear and very few suspension

### 3.2 Method validation

The developed method was validated with important parameters, such as the correlation coefficient, sensitivity (LOD and LOQ), accuracy (percentage recovery), and precision (% relative standard deviation). To evaluate the linearity of the calibration curves, regression analysis was performed. Good results were obtained, as indicated by the correlation coefficient (*R* ^2^) values, which ranged from .9990 to .9999 (Table 2). LODs were calculated based on the standard deviation of the response (Sy) of the curve and the slope of the calibration curve (*S*) (LOD = 3.3*δ*/*b*), where *b* is the slope of the calibration curve and *δ* is the residual standard deviation of the linear regression (Shrivastava & Gubta, 2011), which is summarized in Table 2. The accuracy of the optimized procedure was evaluated by analyzing the digests of spiked samples of mixed teff as representative of the three types of samples. An intermediate standard solution (100 mg/L) was prepared for each of the analytes using a stock solution, which contained 1000 mg/L. A 0.5 g of sample of the mixed teff was used as a representative for testing recovery. The sample was spiked with a standard solution of known concentrations of the target analytes (Table 3). Then, 0.1 and 0.2 mL of intermediate standard solutions (100 mg/L) were spiked with 0.5 g of sample and 20 mg/kg and 40 mg/kg standard solutions, respectively. The spiked and nonspiked samples were digested and examined under similar conditions, as shown in Table 3. Then, 0.1 and 0.2 mL of intermediate standard solutions (100 mg/L) were spiked with 0.5 g of sample and 20 and 40 mg/kg standard solutions, respectively. The spiked and nonspiked samples were digested and examined under similar conditions, as shown in Table 3. The recovery was calculated using the following formula; hence, the percentage recoveries varied between 80.72% and 107.79%, with the majority of analytes falling between 100 ± 10%.

$$\text{Recovery}=\frac{\text{Spiked Sample}-\text{Unspiked Sample}}{\text{Amount added}}\times 100\%$$

**TABLE 2:** Correlation coefficients and sensitivities (limit of detection, LODs and limit of quantification, LOQs) of the optimized digestion method for the teff sample.

Analytes	LOD (mg/kg)	LOQ (mg/kg)	Correlation coefficient (*R* ^2^)
Pb	0.15	0.475	.9993
As	0.028	0.084	.9991
Zn	0.047	0.145	.9999
Cd	0.155	0.47	.9992
Cu	0.039	0.119	.9999
B	0.044	0.133	.9999
Ni	0.252	0.76	.9998
Co	0.289	0.87	.9991
Hg	0.06	0.183	.9998
Fe	0.652	1.976	.9999
Mn	0.036	0.111	.9999
Cr	0.137	0.416	.9989
Ca	4.66	14.12	.9992
K	14.49	43.93	.9980
Na	9.71	29.44	.9991
Mg	5.26	15.95	.9990
P	2.38	7.23	.9998

**TABLE 3:** Recovery of target analytes from teff samples; three replicates and three readings (*n* = 9).

Analytes	Unspiked sample (mg/kg) ±%RSD	Added amount (mg/kg)	Amount detected in the spiked (mg/kg) ±%RSD	Recovery (%)
Zn	21.5 ± 2.7	20	38.6 ± 1.07	85.3
Cu	6.5 ± 8.8	20	24.8 ± 0.7	91.55
Fe	297.1 ± 0.8	40	334.04 ± 2.5	92.35
Mn	125.2 ± 1.5	20	144.2 ± 0.43	95.37
As	0.14 ± 10.3	20	19.9 ± 1.1	98.8
B	3.7 ± 2.33	20	22.4 ± 1.2	93.69
Cd	1.2 ± 0.1	20	19.4 ± 1.6	90.96
Co	BQL	‐	‐	‐
Cr	BQL	‐	‐	‐
Hg	2.56 ± 10.8	20	21.4 ± 1.7	94.12
Ni	2.7 ± 5.5	20	21.4 ± 2.8	93.55
Pb	3.5 ± 3.3	20	22.9 ± 0.5	97.45
Ca	1640.3 ± 1.3	40	1677.2 ± 0.5	92.36
Mg	1440.2 ± 2.4	40	1473.12 ± 0.63	82.32
Na	115.4 ± 9.7	40	147.8 ± 0.12	80.72
K	3097.4 ± 2.1	40	3134.52 ± 0.16	92.8
P	4010.4 ± 1.8	40	4053.516 ± 0.26	107.79

Abbreviation: BQL, below quantification limit.

The percentage relative standard deviations of the triplicate sample preparation and the triplicate reading (*n* = 9) of the three teff samples were used to report the precision of the results. Table 4 shows that good precision was achieved, with a percentage RSD ranging from 0.017% to 11.1%.

**TABLE 4:** Levels of each studied mineral nutrient and nonessential metal (mg/kg in dry weight) and the percentage relative standard deviation (%RSD) of the teff samples (*n* = 9).

Analytes	Red teff	Mixed teff	White teff
	Mean ± %RSD	Mean ± %RSD	Mean ± %RSD
Ca	1930.3 ± 1.3	1625.7 ± 1.59	1918.94 ± 0.7
Mg	1654.6 ± 2.4	1447.2 ± 1.3	1421.64 ± 1.2
Na	54.4 ± 9.6	110.42 ± 1.45	136.12 ± 0.5
K	3696.33 ± 2.17	3108.5 ± 0.023	3474.2 ± 0.48
P	4779.16 ± 0.017	3935.76 ± 1.56	4074.19 ± 0.37
Mn	82 ± 1.5	126.72 ± 1.19	64.461 ± 0.5
Fe	183.33 ± 0.8	300.69 ± 1.7	130.66 ± 1.78
Ni	3.56 ± 9.4	2.95 ± 10.2	2.85 ± 10.6
Co	BQL	BQL	BQL
Cu	4.37 ± 4.5	6.94 ± 0.8	4.63 ± 1.5
Zn	26.62 ± 2.8	20.58 ± 1.1	20.08 ± 1.04
Cd	1.757 ± 0.199	1.32 ± 8	0.992 ± 8.08
Hg	3.667 ± 9.5	2.793 ± 7.5	2.90 ± 3.85
Pb	2.82 ± 8.86	3.447 ± 8.03	1.32 ± 7.8
As	0.029 ± 10.3	0.161 ± 11.1	0.23 ± 8.1
B	21.476 ± 2.3	3.56 ± 7.9	1.943 ± 4.8
Cr	BQL	BQL	BQL

Abbreviations: BQL, below the quantification limit; %RSD, percent relative standard deviation.

### 3.3 Determination of mineral nutrient and nonessential metal concentrations in the teff samples

The level of analytes under study depends on the nature of the crop and geographical location. Nutrient and nonessential metal absorption and transfer from the soil are complex processes that depend on various factors. The degree of heavy metal contamination in the soil, the availability of minerals in soluble and usable forms, the quantity of a given mineral in a given area, the root surface area, the pH, aeration, the temperature, the root surface area, and the ability of plants to absorb metals from the soil are a few examples of heavy metal contamination. The biosphere, the primary source of metals in soil, can be contaminated by large‐scale modern agricultural practices and rapid industrialization. This includes the use of different types of fertilizers, pesticides, herbicides, and other chemicals in the soil. The contamination of heavy metals in crops is a serious threat to food safety and human health. The use of metal‐based pesticides and fertilizers, irrigation with contaminated water, and contaminated soils can trigger the accumulation of heavy metals in plants (Zhang et al., 2021).

Although there was no industrialization in the study area, continuous agrochemical chemical application was identified in Hidabu Abote Woreda, which may contribute to the accumulation of metals in the soil (Abebe et al., 2020; Feyisa et al., 2022). The pH of the soil in Hidabu Abote Woreda has been shown to be somewhat acidic, which may affect the availability of nutrients and plant uptake. Macronutrients such as calcium, magnesium, sulfur, potassium, nitrogen, and potassium are easily accessible at pH values between 6.0 and 6.5; however, at higher pH values (pH > 7.0), the availability of macronutrients decreases (Ferrarezi et al., 2022).

**FIGURE 2:**
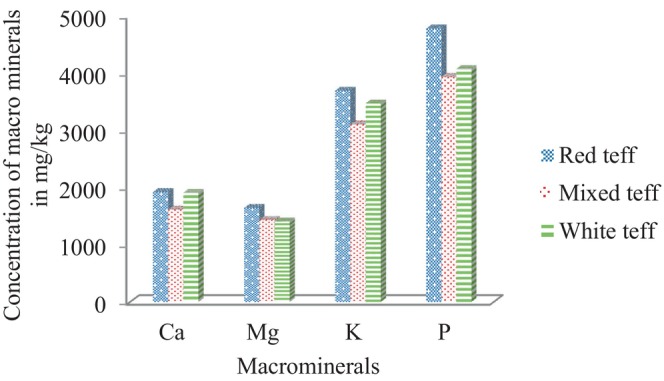
Distribution pattern of the macroelements in the red, mixed, and white teff samples.

**FIGURE 3:**
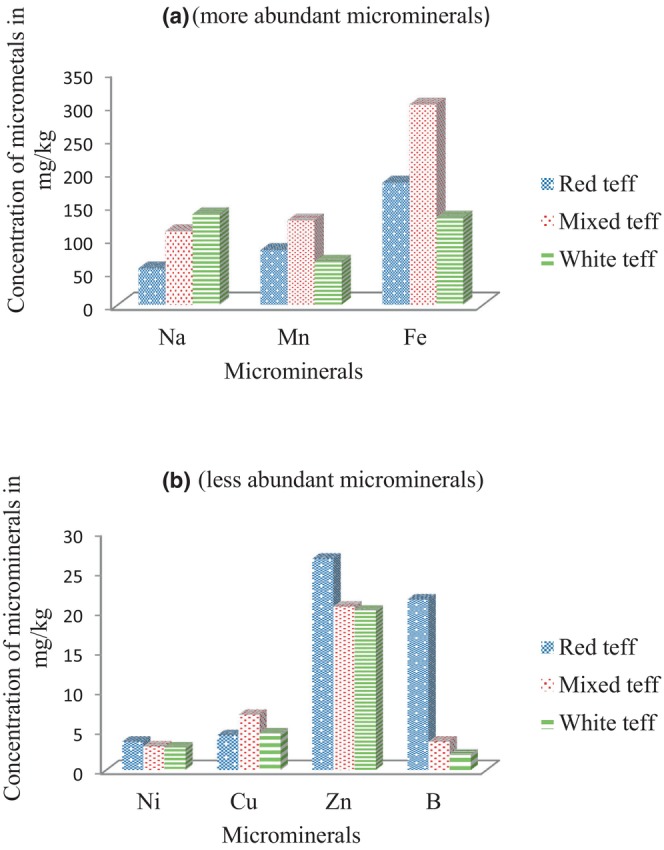
(a, b) Distribution patterns of microelements in the red, mixed, and white teff samples.

**FIGURE 4:**
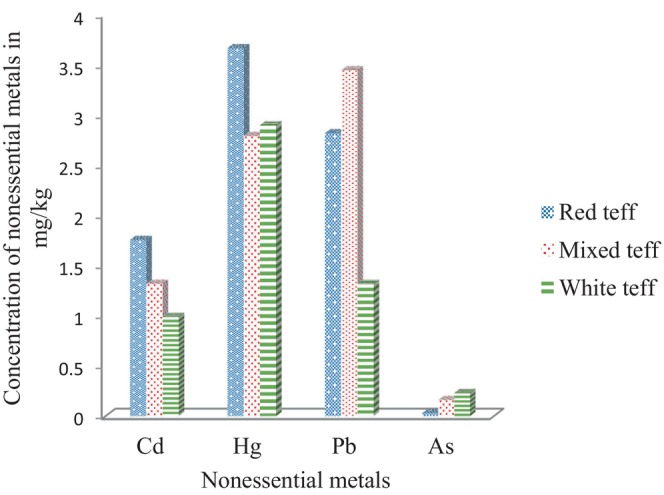
Distribution of toxic element concentrations in red, mixed, and white teff samples.

The concentrations of the examined analytes in the teff samples taken from Hidabu Abote Woreda are shown in Table 4. Figures 2, 3a,b, and 4 show the results. Triple sample preparation and triplicate recording were employed, and the results are presented as the mean values with percentage relative standard deviation (%RSD) in mg/kg.

The white teff sample had higher concentrations of Na and As than did the red and mixed teff samples, while the mixed teff sample had higher concentrations of Mn, Fe, Cu, and Pb. The concentrations of the analytes revealed that the red teff samples had higher concentrations of Ca, Mg, K, P, Ni, Zn, Cd, Hg, and B than did the white and mixed teff samples. As shown in Figure 2, white teff has somewhat greater K, Ca, P, and Mg concentrations than red teff and mixed teff, but overall, the K, Ca, P, and Mg concentrations are the highest (in the top four minerals) of all three samples. As indicated in Table 4 and Figure 2, the concentration of P was identified as the highest of all the analytes investigated throughout a variety of teffs. Its concentration ranged from 3890 to 4853 mg/kg, followed by K (3040–3784 mg/kg), Ca (1906–1956 mg/kg), and Mg (1402–1698 mg/kg). The higher levels of phosphorus, potassium, calcium, and magnesium in teff probably occurred because nutrient elements such as P, K, Ca, and Mg are highly mobile in plant tissue and translocate from old plant tissue to new plant tissue (Chandravanshi et al., 2010). The other probable reason for the higher concentrations of P, K, Ca, and Mg is that the soil used for cultivating the plant is highly fertilized with manure and organic residues, which are high in available phosphorus, potassium, calcium, and magnesium. Hence, plants contain high amounts of these metals (Abebe et al., 2020; Chandravanshi et al., 2010).

As shown in Figure 3a, out of the micromineral, the concentrations of Fe, Mn, and Na were the highest of all the concentrations in the entire three teff samples. As shown in Figure 3b, Ni was found to be the least abundant in all types of teff, while B was detected in mixed and white teff. The most abundant essential trace metal (microelement) was iron (Fe) (128–305 mg/kg), followed by Mn (64–127.8 mg/kg), Na (50–136.5 mg/kg), Zn (19.8–27.3 mg/kg), B (1.8–21.9 mg/kg), Cu (4.17–6.9 mg/kg), and Ni (2.6–4.05 mg/kg). Cr and Co were found to be below the method quantitation limit. The highest concentration of Fe may be attributed to its higher levels in the soil (Yohannes & Chandravanshi, 2015). The concentrations of nonessential heavy metals (toxic) detected in the teff samples were 0.048–3.7 mg/kg for Pb, 0.012–2.09 mg/kg for Cd, 0.02–0.24 mg/kg for As, and 1.8–4.0 mg/kg for Hg. The uptake and accumulation of heavy metals depend on the following factors: the degree of heavy metal contamination in the soil, the capacity of plants to absorb metals from the soil, the availability of minerals in soluble and usable forms, and the abundance of specific minerals in specific places.

In general, the concentrations of analytes in the three varieties of teff studied decreased in the following order: red teff: P > K > Ca > Mg > Fe > Mn > Na > Zn > B > Cu > Hg > Ni > Cd > Pb > As; mixed teff: P > K > Ca > Mg > Fe > Mn > Na > Zn > Cu > B > Pb > Ni > Hg > As > Cd; and white teff: P > K > Ca > Mg > Na > Fe > Mn > Zn > Cu > Ni > Hg > B > Cd > As > Pb. The concentrations of the elements Co and Cr were below the quantitation limits.

### 3.4 Pearson correlation of mineral nutrients and nonessential metals

The impact of one analyte concentration on the concentration of another analyte was correlated in this study using Pearson correlation matrices with a correlation coefficient (*r*) for the samples. The findings are displayed in Table 5. A correlation coefficient of +1.0 indicates a perfect positive correlation, while a correlation coefficient of −1.0 indicates a perfect negative correlation. A moderate correlation occurred (approximately ±0.5) for some other analytes, and a weak correlation existed (<±0.5) for the remaining analytes.

**TABLE 5:** Pearson correlation matrices of mineral nutrients and nonessential metals for teff samples.

	Ca	Mg	Na	K	P	Mn	Fe	Ni	Cu	Zn	Cd	Hg	Pb	As	B
Ca	1														
Mg	.53	1													
Na	−.24	.951	1												
K	.952	.764	−.95	1											
P	.652	.988	.893	.853	1										
Mn	−.95	−.246	−.16	−.81	−.390	1									
Fe	−.94	−.216	−.09	−.79	−.361	1**	1								
Ni	.474	.998**	−.96	.721	.977	−.18	−.15	1							
Cu	−.99	−.57	.287	−.96	−.685	.937	.926	−.52	1						
Zn	.465	.997*	−.97	.714	−.974	−.17	−.14	1**	−.51	1					
Cd	.94	.894	−.99	.394	.816	.214	.244	.921	−.14	.925	1				
Hg	.622	.994	−.91	.832	.999*	−.35	.325	.984	−.66	.982	.838	1			
Pb	−.70	.232	−.52	−.45	.081	.886	.900	.290	.660	.303	.643	.120	1		
As	.882	.867	−.67	.98	.93	−.69	−.67	.83	−.90	.827	.55	.918	−.28	1	
B	.463	.997*	.972	.713	.974	−.17	−.41	1**	−.51	1**	.926	.982	.305	.826	1

** Correlation is significant at the .01 level (two‐tailed).

* Correlation is significant at the .05 level (two‐tailed).

There was a perfect positive correlation between Fe and Mn, between Zn and Ni, and between B and both Ni and Zn. There were strong positive correlations between Ni and Mg; Zn with Mg; Hg with P; B with Mg; Mg with Na; K with Ca; P with Mg and Na; Cu with Mn; and Cd with Ca, Mg, Ni, Zn, and Hg with Ni and Zn. Moderate positive correlations were detected between Cu and Ni; Mg with Ca; Zn with Cu; Hg with Ca; Pb with Cu and Cd; and As with Cd and Ca with P. Weak positive correlations were detected between Mn and Cd and Hg; Pb with Ni, Zn, and Hg; and B with Pb. A moderately negative correlation was observed between Cu and Mg, Ni, and P; Pb, with Na and K; P, with Fe; and B, with Fe and Cu. Weak negative correlations were recognized between Mn and Mg and Na, between Fe and Mg and P, between Ni and Fe, between Zn and Mn and Fe, and between As and Pb. Strong negative correlations were recognized between Ca and Mn, Fe, and Cu; between Na and Zn, Cd, and Hg; and between As and Cu and between P and Zn. The reasons for strong, moderate, and weak correlations between analytes are complex and need further study. There may be many reasons for this: the chemistry of the analytes themselves, the availability of the analyte in the soil, the pH of the soil, the type of soil, and its chemistry (Habte et al., 2020). The availability of a large amount of minerals can reduce the toxicity of toxic metals because the amount of minerals exceeds the amount of toxic metals at the binding site. If harmful metals replace vital minerals due to competitive absorption between the two, health problems could arise.

In this research, several toxic metals were also detected in teff. It is difficult to reduce exposure to toxic metals because soils contain both toxic metals and essential elements. However, high concentrations of minerals help mitigate the load of toxic metals. Research has shown that dietary calcium reduces the risk of lead poisoning by decreasing the absorption of lead through the gastrointestinal tract. Lead and cadmium absorption are increased in individuals with iron deficiency. Lead and iron have antagonistic interactions, which means that iron competes with lead and that enough iron reduces lead toxicity. Cadmium interferes with the metabolism of copper, zinc, and iron (Rădulescu & Lundgren, 2019; Thomas, 2021). In this study, the correlation between the analytes under investigation ranged from strongly positive to strongly negative. Table 5 shows that there were strong positive correlations between Cd and Ca, Mg, Ni, and Zn; between B and Hg; and between Hg and both Ni and Zn. There was no strong negative relationship between toxic metals and minerals except between Na and Hg.

## 4 CONCLUSION AND RECOMMENDATIONS

In this study, ICP‐OES was used to measure the concentrations of nonessential metals (Pb, Cd, Hg, Cr, and As) and mineral nutrients (Ca, Mg, Na, K, P, B, Zn, Cu, Fe, Mn, Co, and Ni) in red, white, and mixed samples taken from Hidabu Abote Woreda in the North Shoa Zone. The overall mean concentrations in the three types of teff samples (mg/kg, in dry weight) were in the order of P (3890–4853) > K (3040–3784) > Ca (1906–1959) > Mg (1402–1698) > Fe (128–305) > Mn (64–127.8) > Na (50–136.5) > Zn (19.8–27.3) > B (1.8–21.9) > Cu (4.17–6) > Ni (2.6–4.05). The results showed that teff grains are a good source of minerals and nutrients. In this study, significant amounts of nonessential elements such as mercury, lead, and copper were also found. The nature of the soil and agronomic practices such as fertilizer and pesticide use may explain this difference, as they may have an impact on the accumulation of harmful metals such as Pb, Hg, and Cd. All three kinds of teff cultivated in Hidabu Abote were shown to have the greatest levels of phosphorus (P), followed by potassium (K), calcium (Ca), and magnesium (Mg). Nevertheless, the three types of teff samples did not contain the studied elements, Co or Cr. Red teff was found to have the lowest concentration of Mn and Fe, while mixed teff samples had the greatest quantities. Teff grains in the research region are often a reliable source of important metals. However, further investigations are needed on the possible sources of the toxic metals Cd, Hg, and Pb.

## AUTHOR CONTRIBUTIONS

**Moges Kebede Seyoum:** Conceptualization (equal); data curation (equal); formal analysis (equal); funding acquisition (lead); investigation (equal); methodology (equal); project administration (equal); resources (lead); validation (equal); visualization (equal); writing – original draft (equal); writing – review and editing (supporting). **Girma Regassa Fayissa:** Conceptualization (equal); data curation (equal); formal analysis (equal); investigation (equal); methodology (equal); project administration (equal); supervision (lead); validation (equal); visualization (lead); writing – original draft (equal); writing – review and editing (lead). **Girma Selale Geleta:** Conceptualization (supporting); data curation (supporting); formal analysis (equal); investigation (equal); methodology (supporting); project administration (equal); supervision (supporting); validation (supporting); visualization (equal); writing – original draft (equal); writing – review and editing (equal).

## CONFLICT OF INTEREST STATEMENT

The authors declare that they have no conflicts of interest.

## ETHICS STATEMENT

This study does not involve any human or animal testing.

## INFORMED CONSENT

Written informed consent was obtained from all study participants.

## Data Availability

The authors confirm that the data supporting the findings of this study are available within the article. Further needed data are available from the corresponding author upon reasonable request.
